# Prognostic significance of diabetes and stress hyperglycemia in acute stroke patients

**DOI:** 10.1186/s13098-022-00896-9

**Published:** 2022-08-29

**Authors:** Antonio Muscari, Roberta Falcone, Guerino Recinella, Luca Faccioli, Paola Forti, Marco Pastore Trossello, Giovanni M. Puddu, Luca Spinardi, Marco Zoli

**Affiliations:** 1 Stroke Unit, Medical Department of Continuity of Care and Disability, IRCCS Azienda Ospedaliero-Universitaria di Bologna, Bologna, Italy; 2 Department of Medical and Surgical Sciences, University of Bologna, Via Massarenti, 9, 40138 Bologna, Italy; 3 Diagnostic and Interventional Neuroradiology Unit, IRCCS Azienda Ospedaliero-Universitaria di Bologna, Bologna, Italy

**Keywords:** Diabetes, Outcome, Prognosis, Stress hyperglycemia, Stroke

## Abstract

**Background:**

Hyperglycemic non-diabetic stroke patients have a worse prognosis than both normoglycemic and diabetic patients. Aim of this study was to assess whether hyperglycemia is an aggravating factor or just an epiphenomenon of most severe strokes.

**Methods:**

In this retrospective study, 1219 ischemic or hemorrhagic stroke patients (73.7 ± 13.1 years) were divided into 4 groups: 0 = non-hyperglycemic non-diabetic, 1 = hyperglycemic non-diabetic, 2 = non-hyperglycemic diabetic and 3 = hyperglycemic diabetic. Hyperglycemia was defined as fasting blood glucose ≥ 126 mg/dl (≥ 7 mmol/l) measured the morning after admission, while the diagnosis of diabetes was based on a history of diabetes mellitus or on a glycated hemoglobin ≥ 6.5% (≥ 48 mmol/mol), independently of blood glucose levels. All diabetic patients, except 3, had Type 2 diabetes. The 4 groups were compared according to clinical history, stroke severity indicators, acute phase markers and main short term stroke outcomes (modified Rankin scale ≥ 3, death, cerebral edema, hemorrhagic transformation of ischemic lesions, fever, oxygen administration, pneumonia, sepsis, urinary infection and heart failure).

**Results:**

Group 1 patients had more severe strokes, with larger cerebral lesions and higher inflammatory markers, compared to the other groups. They also had a high prevalence of atrial fibrillation, prediabetes, previous stroke and previous arterial revascularizations. In this group, the highest frequencies of cerebral edema, hemorrhagic transformation, pneumonia and oxygen administration were obtained. The prevalence of dependency at discharge and in-hospital mortality were equally high in Group 1 and Group 3. However, in multivariate analyses including stroke severity, cerebral lesion diameter, leukocytes and C-reactive protein, Group 1 was only independently associated with hemorrhagic transformation (OR 2.01, 95% CI 0.99–4.07), while Group 3 was independently associated with mortality (OR 2.19, 95% CI 1.32–3.64) and disability (OR 1.70, 95% CI 1.01–2.88).

**Conclusions:**

Hyperglycemic non-diabetic stroke patients had a worse prognosis than non-hyperglycemic or diabetic patients, but this group was not independently associated with mortality or disability when size, severity and inflammatory component of the stroke were accounted for.

## Background

There is no doubt about the importance of diabetes as a stroke risk factor. Instead, what is still controversial is the role played by diabetes and hyperglycemia on the outcome of acute stroke. From the 90 s to present days, the subject has been treated in numerous publications, without reaching definitive conclusions. The most interesting and intriguing result is the almost generalized finding of a worse prognosis in hyperglycemic non-diabetic patients than in diabetic patients [1–13].

These observations led to the definition of “stress hyperglycemia” as a possible important factor worsening the prognosis of stroke patients [14]. In fact, experimental studies suggest that an excess of glucose may be detrimental to brain structures and metabolism [15]. In particular, as illustrated in an excellent review by Li et al. [16], hyperglycemia intensifies the inflammatory response, resulting in increased permeability of the blood–brain barrier, edema and hemorrhage. In addition, under conditions of ischemic anaerobiosis, glycemic excess intensifies anaerobic glycolysis, and consequently lactic acidosis. Finally, neuronal damage can result from the accumulation of calcium and reactive oxygen species, and from the vasoconstrictor effect caused by a lower availability of nitric oxide.

Despite these documented harmful effects of hyperglycemia, uncertainty remains as to whether the hyperglycemia associated with severe strokes is a contributing factor to such severity, or just an epiphenomenon of severity. This distinction is of considerable practical relevance: in fact, in the first case it would be important to try to control, even aggressively, hyperglycemia even in non-diabetic patients, while in the second case such control would be unnecessary, if not potentially harmful. To shed light on this topic, it is important to correct the statistical analysis for all the main confounding factors: age, sex, acute phase markers, stroke severity and cerebral lesion size. These factors have not always been considered in the various studies and, in particular, the extent of brain injury has almost never been considered.

The present study aims to bring a new contribution to the understanding of the relationships between diabetes, stress hyperglycemia and acute stroke outcomes. The analysis was corrected for the main confounding factors associated with stroke severity and inflammatory status, also considering, in particular, cerebral lesion diameter.

## Materials and methods

### Patients

This retrospective observational cross-sectional study included all patients admitted to our stroke unit, for acute ischemic or hemorrhagic stroke, from January 19, 2011, to December 30, 2015 (N = 1233). Patients whose fasting blood glucose was not available (N = 14) were excluded from the study. Thus, the sample used for the analyses included 1219 patients with a mean age of 73.7 ± 13.1 years, 624 of whom (51.2%) were men.

In accordance with the American Diabetes Association (ADA) and the International Expert Committee on HbA1c (IEC HbA1c) [17, 18], patients with a history of diabetes mellitus or with glycated hemoglobin ≥ 6.5% (≥ 48 mmol/mol) were considered diabetic.

Hyperglycemia was defined as fasting blood glucose ≥ 126 mg/dl (≥ 7 mmol/l) measured the morning after admission. In order to study the independent contribution of high glucose levels, which are often present in the acute phase of stroke, to the short-term prognosis, hyperglycemia, even if detected in more than one measurement, was not considered sufficient for the diagnosis of diabetes, if not associated with increase in glycated hemoglobin or with previous diagnosis of diabetes.

In this way, patients were divided into 4 groups: Group 0 = non-hyperglycemic non-diabetic (N = 884), Group 1 = hyperglycemic non-diabetic (N = 73), Group 2 = non-hyperglycemic diabetic (N = 101), and Group 3 = hyperglycemic diabetic (N = 161). Almost all diabetic patients had Type 2 diabetes: there were only 3 cases of Type 1 diabetes (2 in Group 2 and 1 in Group 3).

In relation to etiology, ischemic strokes (N = 1055) were classified into 5 categories according to the Trial of ORG 10172 in Acute Stroke Treatment (TOAST) criteria [19]. In relation to the site and extent of the lesion, ischemic strokes were clinically classified as lacunar syndromes (LACS), partial anterior circulation syndromes (PACS), total anterior circulation syndromes (TACS) and posterior circulation syndromes (POCS), according to the Oxfordshire Community Stroke Project (OCSP) criteria [20]. In relation to the location, hemorrhagic strokes (N = 164) were classified as “atypical lobar” or “typical deep” (the latter including all non-lobar sites). Stroke severity on stroke unit admission was described using the National Institutes of Health Stroke Scale (NIHSS) score [21]. The degree of disability upon discharge from the stroke unit was described using the modified Rankin scale (mRS) [22], and a score ≥ 3 (loss of autonomy) was considered relevant. Short-term mortality from any cause was ascertained during stroke unit stay (median duration: 7 days). Fever was defined as a temperature ≥ 37.5 °C (temperature was measured at least 3 times a day). Oxygen was administered when arterial oxygen saturation (measured at least once a day) was ≤ 92% [23]. Present or previous atrial fibrillation was defined based on clinical history and continuous ECG monitoring.

Because this was a retrospective study including many patients deceased after the stroke, an informed consent could not be obtained. However, at the time of hospitalization patients were informed that their data could be used for research purposes and the study protocol was approved by our Hospital-University Ethics Committee.

### Laboratory and instrumental variables

Blood glucose, leukocyte count and high-sensitivity C-reactive protein were measured on fasting venous blood sampling performed the morning after admission. Glycated hemoglobin was measured by high performance liquid chromatography (HPLC) in diabetic patients and those with high blood glucose (≥ 126 mg/dl or 7 mmol/l). All ischemic stroke patients underwent ultrasonography of supraortic trunks, and carotid stenosis ≥ 50%, both ipsi- and contralateral, was recorded. Patients underwent at least 2 brain CT scans: the first on admission to the Emergency Department and the second on average on the third day of hospitalization. From the images of the second brain CT, the maximum diameter in cm of the brain lesion, both ischemic and hemorrhagic, was obtained, as well as the possible presence of cerebral edema, defined as a hypodense area with mass effect. Hemorrhagic transformation of the ischemic area was defined as any of the 4 levels of the European Cooperative Acute Stroke Study (ECASS) scale [24], which was detected in any of the brain CT scans performed during hospitalization.

### Statistical analysis

The continuous variables with Gaussian distribution were described with mean and standard deviation and compared with analysis of variance (ANOVA) or Student’s t test for unpaired data. The continuous variables with non-Gaussian distribution were described with median and interquartile range and compared with Kruskal–Wallis’ and Mann–Whitney’s tests. Percentages were compared with χ^2^ test.

Survival curves were obtained with Kaplan Meier analysis and compared with log rank test.

Multivariate analysis for the dependent variables cerebral edema, hemorrhagic transformation, fever, pneumonia, oxygen administration and relevant disability (mRS ≥ 3) was performed by logistic regression, which produced odds ratios and relative confidence intervals. For the dependent variable death, Cox regression was performed, which produced hazard ratios. The possible presence of collinearity in the models was assessed by Variance Inflation Factor (VIF) analysis. The matrix of multivariate correlations between blood glucose, acute phase markers and stroke severity indicators was carried out by multiple linear regressions, after logarithmic transformation of the variables to obtain their normalization, and backward elimination of non-significant associations was performed, so that all residual variables were independently and significantly associated with the dependent variable.

Two-tail tests were performed throughout, and P-values < 0.05 were considered significant. For statistical calculations, SPSS v. 22 software was used (IBM, Armonk, New York, USA).

## Results

### General characteristics of the 4 groups of stroke patients

Table 1 shows the main characteristics of the 4 groups of stroke patients (1055 ischemic and 164 hemorrhagic). Diabetic patients, both non-hyperglycemic and hyperglycemic, had higher glucose levels than the corresponding non-diabetic groups. The hyperglycemia of non-diabetic patients (Group 1) was generally mild and transient, falling below the threshold of 126 mg/dl (7 mmol/l) within a few days and, in fact, only in 9.6% of cases a short insulin treatment was administered. In sporadic cases (1.9%) insulin was administered to Group 0 patients who had hyperglycemia in the days following admission. Glycated hemoglobin values were highest in Group 3, intermediate in Group 2 and lowest in Group 1 (glycated hemoglobin was not measured in Group 0). Among group 1 patients, 46.7% had glycated hemoglobin ≥ 6% (≥ 42 mmol/mol), thus being defined as high risk prediabetic according to the International Expert Committee on HbA1c [18]. Compared to Group 0, in Group 3 there was a higher prevalence of men and chronic kidney disease; in addition, in both groups of diabetic patients there was a higher frequency of hypertension, hypertriglyceridemia, HDL hypocholesterolemia, carotid stenosis, previous myocardial infarction, peripheral artery disease and arterial revascularizations. In relation to all these variables, Group 1 did not differ significantly from Groups 2 and 3, and generally had higher values, although not significantly, than Group 0. In addition, in Group 1 there was a higher frequency of atrial fibrillation than in Groups 0 and 3, a higher frequency of previous stroke than in Group 3 and a higher frequency of arterial revascularizations than in Group 0. Finally, Group 1 had a significantly lower prevalence of small artery strokes than all other groups, while Group 3 had the highest prevalence of such strokes and the lowest frequency of thrombolysis.

**Table 1:** Characteristics of the four groups (N = 1219, 1055 ischemic and 164 hemorrhagic strokes)

	Group				P value
	0 (N = 884)	1 (N = 73)	2 (N = 101)	3 (N = 161)	
General characteristics					
Blood glucose (mmol/l)	4.89 [4.39–5.50]	7.44 [7.17–8.00]	5.83 [5.11–6.33]^d0^	9.44 [8.11–11.67]^d1^	–
In-hospital insulin treatment	17 (1.9)	7 (9.6)^d0^	58 (57.4)^d0d1^	131 (81.4)^d0d1d2^	< 0.0001
HbA1c (%)	–	5.9 [5.6–6.2]	6.8 [6.3–7.4]	7.6 [6.9–8.5]^d2^	–
Age (years)	73.7 ± 13.7	73.2 ± 12.8	74.2 ± 10.6	73.5 ± 10.7	0.97
Male sex	427 (48.3)	41 (56.2)	57 (56.4)	99 (61.5)^b0^	0.009
Hypertension	730 (82.6)	64 (87.7)	94 (93.1)^b0^	147 (91.3)^b0^	0.002
Hypercholesterolemia	567 (64.1)	40 (54.8)	66 (65.3)	98 (60.9)	0.37
Hypertriglyceridemia	60 (6.8)	9 (12.3)	14 (13.9)^b0^	36 (22.4)^d0^	< 0.0001
HDL Hypocholesterolemia	113 (12.8)	13 (17.8)	20 (19.8)^a0^	40 (24.8)^d0^	0.0005
Ever smoker	365 (41.3)	31 (42.5)	49 (48.5)	71 (44.1)	0.54
Alcohol drinker	155 (17.5)	14 (19.2)	14 (13.9)	21 (13.0)	0.41
Carotid stenosis ≥ 50%*	232 (30.6)	26 (43.3)	45 (48.9)^c0a3^	48 (33.3)	0.002
Atrial fibrillation	277 (31.3)	31 (42.5)^a0a3^	36 (35.6)	45 (28.0)	0.13
Chronic kidney disease	79 (8.9)	8 (11.0)	14 (13.9)	30 (18.6)^c0^	0.002
Previous myocardial infarction	95 (10.7)	12 (16.4)	26 (25.7)^d0^	36 (22.4)^d0^	< 0.0001
Previous stroke	112 (12.7)	14 (19.2)^a3^	23 (22.8)^b0b3^	15 (9.3)	0.006
Peripheral artery disease	39 (4.4)	4 (5.5)	14 (13.9)^d0^	15 (9.3)^b0^	0.0004
Previous arterial revascularizations	65 (7.4)	12 (16.4)^b0^	19 (18.8)^d0^	23 (14.3)^b0^	< 0.0001
TOAST classification*					
Large artery	125 (16.5)	10 (16.7)	22 (23.9)	25 (17.4)	0.36
Cardioembolism	228 (30.0)	22 (36.7)	26 (28.3)	40 (27.8)	0.63
Small artery	136 (17.9)^a1^	4 (6.7)	15 (16.3)	44 (30.6)^c0c1b2^	0.0002
Other determined	22 (2.9)	3 (5.0)	4 (4.3)	2 (1.4)	0.43
Undetermined	248 (32.7)	21 (35.0)	25 (27.2)	33 (22.9)	0.09
i.v. Thrombolysis*	135 (17.8)	9 (15.0)	14 (15.2)	14 (9.7)^a0^	0.11
Stroke severity indicators					
NIHSS score	7 [3–14]	12 [6–18]^d0c2a3^	6 [3–14]	8 [4–14]^a0^	< 0.0001
Cerebral lesion diameter (cm)	2.7 [1.1–4.7]	4.2 [1.8–6.7]^d0c2a3^	2.3 [0.9–4.6]	2.4 [0.9–4.9]	0.005
OCSP classification*					
LACS	171 (22.5)	4 (6.7)^a0b2b3^	24 (26.1)	37 (25.7)	0.02
PACS	305 (40.2)	27 (45.0)	36 (39.1)	44 (30.6)	0.13
TACS	163 (21.5)	22 (36.6)^b0^	22 (23.9)	35 (24.3)	0.06
POCS	120 (15.8)	7 (11.7)	10 (10.9)	28 (19.4)	0.27
Hemorrhagic stroke	125 (14.1)	13 (17.8)	9 (8.9)	17 (10.6)	0.21
“Typical” deep location	81 (64.8)	6 (46.2)	6 (66.7)	13 (76.5)	0.39
“Atypical” lobar location	44 (35.2)	7 (53.8)	3 (33.3)	4 (23.5)	0.39
Acute phase markers					
Leukocytes (10^9^/l)	8.26 [6.77–10.16]	9.95 [8.11–12.71]^d0c2a3^	8.43 [6.88–10.31]	9.43 [7.46–11.16]^c0^	< 0.0001
C-reactive protein (mg/dl)	0.77 [0.29–2.52]	1.72 [0.65–4.51]^c0c2c3^	0.63 [0.32–2.22]	0.82 [0.35–2.35]	0.002
Fever	250 (28.3)	35 (47.9)^c0a2a3^	32 (31.7)	59 (36.6)	0.002
Outcomes					
Modified Rankin scale ≥ 3	624 (70.6)	62 (84.9)^b0b2^	69 (68.3)	131 (81.4)^b0a2^	0.002
Death	48 (5.4)	10 (13.7)^b0^	8 (7.9)	25 (15.5)^d0^	< 0.0001
Cerebral edema	317 (35.9)	42 (57.5)^c0d2c3^	29 (28.7)	61 (37.9)	0.0008
Hemorrhagic transformation*	139 (18.3)	25 (41.7)^d0b2c3^	18 (19.6)	26 (18.1)	0.0002
Fever	250 (28.3)	35 (47.9)^c0a2a3^	32 (31.7)	59 (36.6)	0.002
Oxygen administration	187 (21.2)	30 (41.1)^d0b2^	23 (22.8)	53 (32.9)^c0^	< 0.0001
Pneumonia	41 (4.6)	8 (11.0)^a0^	6 (5.9)	7 (4.3)	0.12
Sepsis	26 (2.9)	5 (6.8)	5 (5.0)	7 (4.3)	0.24
Urinary infection	82 (9.3)	6 (8.2)	8 (7.9)	8 (5.0)	0.35
Heart failure	48 (5.4)	8 (11.0)^a0^	7 (6.9)	11 (6.8)	0.26

Values are mean ± SD or median [25th–75th percentile] or number (percentage)

a = P < 0.05, b = P ≤ 0.01, c = P ≤ 0.001, d = P ≤ 0.0001 (number after symbol indicates reference group)

Groups: 0 = nondiabetic nonhyperglycemic, 1 = nondiabetic hyperglycemic, 2 = diabetic nonhyperglycemic, 3 = diabetic hyperglycemic

*HbA1c* Glycated hemoglobin, *HDL* high density lipoprotein, *LACS* lacunar syndrome, *NIHSS* National Institutes of Health Stroke Scale, *OCSP* Oxfordshire Community Stroke Project, *PACS* partial anterior circulation syndrome, *POCS* posterior circulation syndrome, *TACS* total anterior circulation syndrome, *TOAST* Trial of Org 10172 in Acute Stroke Treatment

^*^Percentages referred to ischemic strokes only

### Stroke severity indicators and acute phase markers

Group 1 included the most severe strokes. In fact, NIHSS score in this group was by far the highest, and cerebral lesion diameter was by far the largest, compared to the other 3 groups. In addition, the frequency of lacunar syndromes (LACS) was the lowest and the frequency of total anterior circulation syndromes (TACS) was the highest (the latter differed significantly only from Group 0). However, the frequency of hemorrhagic strokes was not significantly different in the 4 groups. In Group 1, the maximum values of acute phase markers were also detected: leukocytes, C-reactive protein and fever prevalence were significantly higher than in the other 3 groups. In Group 3 leukocytes were higher than in Group 0.

### Stroke outcomes

**Fig. 1:**
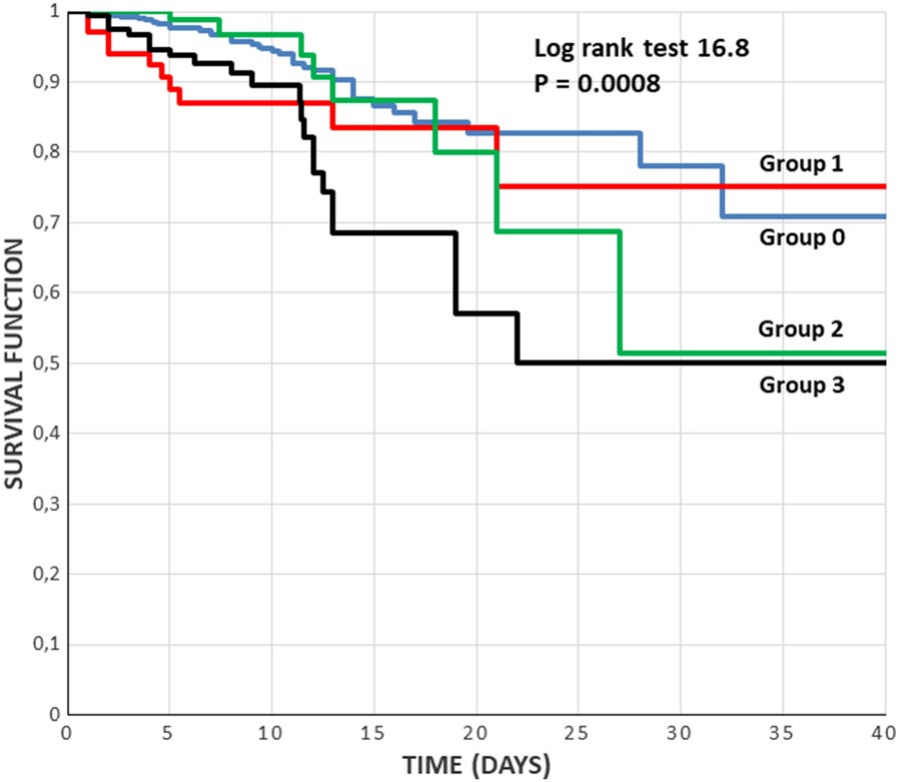
Survival curves of the 4 groups. Groups: 0 = nondiabetic nonhyperglycemic, 1 = nondiabetic hyperglycemic, 2 = diabetic nonhyperglycemic, 3 = diabetic hyperglycemic

Probably due to the greater stroke severity and the more intense acute phase, Group 1 had the highest prevalence of the main outcomes. At discharge, the prevalence of patients with an mRS score ≥ 3 was significantly higher than in Groups 0 and 2, and mortality was significantly higher than in Group 0. Similar results to these were also present in Group 3. However, the survival curves of the 4 groups (Fig. 1) show that Group 3 had the worst course, while the temporal course of Group 1 was similar to that of Group 0.

In addition, in Group 1 there was the maximum prevalence, significantly higher than in the other 3 groups, of cerebral edema, hemorrhagic transformation and fever (the latter already considered among acute phase indicators). In Group 1, the need for oxygen supplementation was greater than in Groups 0 and 2, and the frequency of pneumonia and heart failure was higher than in Group 0.

### Multivariate analysis of main outcomes

Table 2 shows the multivariate analysis of the main outcomes: mRS ≥ 3, death, cerebral edema, hemorrhagic transformation, fever, pneumonia and need of oxygen administration. These 7 outcomes, in the form 1/0 (yes/no), were the dependent variables in 7 multivariate analyses. The possible contribution to these outcomes of Groups 1, 2 and 3 (each of them in the form yes/no) was evaluated also taking into account the main indicators of stroke severity (NIHSS score and cerebral lesion diameter), acute phase markers (leukocytes, C-reactive protein and fever), cardiovascular events in past medical history (myocardial infarction, stroke, peripheral artery disease and arterial revascularizations), plus age and sex, for a total of 14 independent variables. The analysis of each outcome included all 14 independent variables, except fever when the outcome was fever. The maximum VIF value was 1.85 (concerning NIHSS score when hemorrhagic transformation was the dependent variable), well below the limit of 10 over which collinearity is probably present. Overall, outcomes were mainly associated with age, stroke severity and acute phase markers. Group 1 remained associated with hemorrhagic transformation with borderline significance, Group 2 did not remain independently associated with any outcomes, and Group 3 remained independently associated with mortality (but the strongest predictor of death was NIHSS score) and, with lower significance, with disability and oxygen administration.

**Table 2:** Multivariate analysis of outcome predictors (N = 1182)

Variables	Outcomes						
	mRS ≥ 3 (N = 856)	Death (N = 89)	Cerebral edema (N = 434)	Hemorrhagic transformation* (N = 203)	Fever (N = 361)	Pneumonia (N = 57)	Oxygen administration (N = 279)
Age (years)	**1.05 [1.03–1.06] < 0.0001**	**1.03 [1.01–1.06] 0.009**	0.99 [0.98–1.01] 0.29	0.99 [0.98–1.01] 0.47	1.01 [0.99–1.02] 0.37	**1.05 [1.01–1.08] 0.006**	**1.04 [1.03–1.06] < 0.0001**
Male sex	1.05 [0.73–1.50] 0.78	**1.65 [1.05–2.58] 0.03**	1.12 [0.79–1.59] 0.51	**1.48 [1.00–2.18] 0.048**	0.94 [0.70–1.26] 0.68	**2.75 [1.45–5.22] 0.002**	1.23 [0.87–1.74 0.25
NIHSS score	**1.40 [1.32–1.48] < 0.0001**	**1.09 [1.05–1.13] < 0.0001**	**1.03 [1.00–1.06] 0.055**	1.01 [0.98–1.04] 0.55	**1.09 [1.06–1.11] < 0.0001**	**1.06 [1.01–1.11] 0.01**	**1.10 [1.07–1.13] < 0.0001**
Cerebral lesion diameter (cm)	**1.12 [1.02–1.23] 0.02**	1.01 [0.95–1.08] 0.73	**2.04 [1.86–2.25] < 0.0001**	**1.47 [1.36–1.58] < 0.0001**	**1.09 [1.03–1.15] 0.002**	1.04 [0.95–1.15] 0.37	1.03 [0.97–1.09] 0.38
Leukocytes (10^9^/l)	1.06 [0.99–1.13] 0.10	**1.07 [1.02–1.13] 0.01**	1.05 [0.99–1.11] 0.09	1.00 [0.94–1.07] 0.95	**1.06 [1.01–1.11] 0.01**	1.07 [0.99–1.15] 0.09	**1.07 [1.02–1.12] 0.006**
C-reactive protein (mg/dl)	1.03 [0.96–1.11] 0.40	1.01 [0.98–1.03] 0.55	0.97 [0.93–1.01] 0.16	**0.95 [0.92–0.99] 0.009**	**1.07 [1.03–1.11] 0.0004**	1.01 [0.97–1.06] 0.51	**1.10 [1.06–1.15] < 0.0001**
Fever	1.46 [0.90–2.37] 0.13	1.36 [0.83–2.22] 0.23	**2.11 [1.46–3.07] < 0.0001**	1.41 [0.94–2.11] 0.10	–	**6.11 [2.99–12.50] < 0.0001**	**2.34 [1.66–3.28] < 0.0001**
Prev. myocardial infarction	1.37 [0.68–2.77] 0.38	1.19 [0.60–2.36] 0.62	**0.43 [0.23–0.84] 0.01**	1.07 [0.57–2.01] 0.83	1.23 [0.76–2.02] 0.40	**2.32 [1.03–5.24] 0.04**	1.36 [0.79–2.36] 0.27
Previous stroke	1.37 [0.80–2.33] 0.25	1.01 [0.56–1.81] 0.98	0.80 [0.48–1.34] 0.40	0.95 [0.55–1.62] 0.84	0.98 [0.65–1.46] 0.91	1.11 [0.50–2.43] 0.80	0.80 [0.50–1.29] 0.36
Peripheral artery disease	**2.80 [1.14–6.89] 0.03**	1.37 [0.61–3.05] 0.45	0.88 [0.41–1.90] 0.75	1.16 [0.57–2.36] 0.69	**0.46 [0.24–0.88] 0.02**	0.88 [0.28–2.75] 0.83	1.22 [0.64–2.35] 0.54
Prev. arterial revascularizations	**0.41 [0.19–0.87] 0.02**	0.55 [0.24–1.27] 0.16	1.72 [0.84–3.52] 0.14	1.37 [0.68–2.78] 0.38	0.86 [0.49–1.52] 0.60	1.04 [0.41–2.66] 0.94	**0.51 [0.26–0.98] 0.04**
Group 1	1.25 [0.52–3.00] 0.61	1.45 [0.71–2.95] 0.30	1.11 [0.53–2.32] 0.78	**2.01 [0.99–4.07] 0.053**	1.55 [0.87–2.74] 0.13	1.26 [0.48–3.33] 0.64	1.77 [0.95–3.32] 0.07
Group 2	0.83 [0.45–1.53] 0.55	1.29 [0.58–2.87] 0.54	0.65 [0.34–1.24] 0.19	1.07 [0.56–2.06] 0.83	1.48 [0.90–2.45] 0.12	1.29 [0.46–3.60] 0.62	1.33 [0.74–2.41] 0.35
Group 3	**1.70 [1.01–2.88] 0.046**	**2.19 [1.32–3.64] 0.003**	0.97 [0.57–1.63] 0.90	0.71 [0.39–1.27] 0.24	1.36 [0.90–2.05] 0.14	0.50 [0.20–1.29] 0.15	**1.73 [1.08–2.78] 0.02**

Results of multiple logistic regression models (Cox regression was performed when the outcome was Death). The numbers in each cell are odds ratio (hazard ratio when the outcome was Death), [95% confidence interval] and P value

Groups: 1 = nondiabetic hyperglycemic, 2 = diabetic nonhyperglycemic, 3 = diabetic hyperglycemic

mRS = modified Rankin scale, NIHSS = National Institutes of Health Stroke Scale

^*^Analysis referred to ischemic strokes only (N = 1027)

Thus, decompensated diabetes was associated with adverse outcomes independently of stroke severity and its inflammatory component, while these adverse outcomes did not seem to be directly associated with Group 1, with the only possible exception of hemorrhagic transformation.

### Relationships among blood glucose, indicators of stroke severity and markers of inflammation in non-diabetic patients

To illustrate the interrelationships in non-diabetic patients (Groups 0 and 1) among blood glucose, indicators of stroke severity and markers of inflammation, Table 3 shows the matrix of multivariate correlations among these factors. Blood glucose, as a dependent variable, was associated with leukocytes and cerebral lesion diameter, while leukocytes, in the role of dependent variable, were strongly associated with blood glucose. However, when cerebral lesion diameter and NIHSS score were the dependent variables, blood glucose was not associated with them.

**Table 3:** Multivariate correlation matrix of blood glucose, acute phase indicators and stroke severity indicators in nondiabetic patients (N = 928)

	Dependent variables				
	Log glucose	Log leukocytes	Log CRP	Log NIHSS	Log CLD
Log glucose	–	0.12 (P = 0.0001)	–	–	–
Log leukocytes	0.15 (P < 0.0001)	–	0.23 (P < 0.0001)	0.10 (P = 0.002)	0.10 (P = 0.003)
Log CRP	–	0.23 (P < 0.0001)	–	0.17 (P < 0.0001)	0.11 (P = 0.0006)
Log NIHSS	–	0.10 (P = 0.004)	0.18 (P < 0.0001)	–	0.30 (P < 0.0001)
Log CLD	0.09 (P = 0.009)	0.09 (P = 0.006)	0.11 (P = 0.0006)	0.29 (P < 0.0001)	–
R^2^	0.03	0.12	0.14	0.17	0.15

Final results of multiple linear regressions after backward elimination of nonsignificant correlations. The numbers in each cell are Pearson’s r correlation coefficients and P values

*CLD* cerebral lesion diameter, *CRP* C-reactive protein, *NIHSS* National Institutes of Health Stroke Scale

## Discussion

This study has confirmed that the most severe strokes mainly occur in the group of hyperglycemic non-diabetic patients [14, 25, 26]. This was reflected in a high frequency of all major outcomes, starting from disability and death (with frequencies comparable to those of hyperglycemic diabetic patients), up to cerebral edema, hemorrhagic transformation and need of oxygen administration (with higher frequencies than in all other groups). However, this study has shown that belonging to Group 1 was not independently associated with the main outcomes of mortality and disability, when indicators of stroke severity and inflammatory status were taken into account, and only the association of Group1 with hemorrhagic transformation was confirmed in multivariate analysis. There are also some pre-stroke characteristics, such as the high prevalence of high-risk prediabetes, arterial revascularizations and atrial fibrillation, which could explain why Group 1 was characterized by strokes of greater severity. Thus, hyperglycemia seems to be a consequence of the increased severity, rather than its possible cause. In addition, neither hyperglycemia nor diabetes seem to affect the occurrence of hemorrhagic strokes, as hemorrhagic strokes were not differently distributed in the 4 study groups.

The following discussion seeks to provide answers to the main questions concerning the relationships among hyperglycemia, diabetes and stroke.

### What is the real contribution of hyperglycemia to the various outcomes in non-diabetic patients?

Our multivariate analysis has shown that there was no independent association of Group 1 with the main outcomes (mortality and disability), and also with cerebral edema, fever and oxygen administration.

In addition to age, the main factors independently associated with all outcomes were NIHSS score and cerebral lesion diameter. It seems unlikely that hyperglycemia in Group 1 may have preceded the stroke and thus contributed to these factors. In fact, hyperglycemia in Group 1 was almost always rapidly reversible, most often without the need for insulin treatment. In addition, in Group 3 hyperglycemia was significantly higher and presumably preceded the stroke, but NIHSS score and cerebral lesion diameter were lower than in Group 1. Finally, in the matrix of multivariate correlations performed in non-diabetic patients, blood glucose was dependent on cerebral lesion diameter and leukocytes, while cerebral lesion diameter and NIHSS score were not dependent on blood glucose.

Another factor associated with many outcomes was inflammation (C-reactive protein, leukocytes and fever). To a large extent, this inflammation could be related to the size of cerebral lesion and stroke severity [27]. Other causes could be the infectious complications (especially pneumonia) that are often associated with most severe strokes. It is known that inflammatory cytokines, by binding to insulin receptors, can induce insulin resistance, and thus contribute to “stress hyperglycemia” [28–31].

It is also possible a reverse mechanism, namely a pro-inflammatory action of hyperglycemia [32–34] and, with the intermediation of inflammation, hyperglycemia could promote hemorrhagic transformation. In fact, inflammation can increase blood–brain barrier permeability and can damage the white matter [16, 35]. An association between hyperglycemia and hemorrhagic transformation had previously been found by others [36] and also by us, previously [37] and in the present study. However, the factor by far most associated with hemorrhagic transformation is the size of cerebral lesion [37], and this may partly explain why we did not find a direct association with hemorrhagic transformation in Group 3, despite the fact that glucose levels in that group were even higher than in Group 1. In fact, in Group 3 there was a high prevalence of lacunar strokes, in accordance with previous observations that diabetes, together with hypertension, is a factor favoring small vessel disease [38]. So, despite the marked hyperglycemia, the high prevalence of small lacunar infarcts in Group 3 made hemorrhagic transformation unlikely.

### What causes hyperglycemia in Group 1?

The most probable cause of hyperglycemia, generally transient, characterizing Group 1, was probably the marked acute phase, witnessed by the increase in the indices of inflammation, in turn an expression of the severity and extent of cerebral lesions. There are various mechanisms by which hyperglycemia can follow the acute phase. It has already been mentioned that inflammatory cytokines, by binding to insulin receptors, may cause hyperglycemia. The main mechanism seems to be reduced tyrosine kinase activity of the insulin receptor induced by tumor necrosis factor-alpha [28]. Another important mechanism linking the acute phase with hyperglycemia is the secretion of adrenal stress hormones. In this regard, if in acute stroke patients van Kooten et al. [39] found no correlation between norepinephrine levels and blood glucose, Tracey et al. [3] found a correlation between cortisol levels and cerebral lesion volume, so that the relationship between blood glucose and mortality disappeared by adjusting the analysis for cortisol levels. Finally, a further possible cause of hyperglycemia is the presence, among non-diabetic patients, of high risk prediabetic patients (about 50% in Group 1), who may be particularly likely to develop hyperglycemia in stressful conditions. In this regard, in a group of 106 patients with ischemic stroke, Vancheri et al. [25] found that more than two-thirds of them had impaired glucose tolerance, even 3 months after the acute stroke.

The presence, in Group 1, of high risk prediabetic patients, could also explain the higher prevalence, compared to Group 0, of atherosclerotic lesions of large vessels, as documented by the significant higher frequency of arterial revascularizations and (although not significantly) of carotid stenosis, previous myocardial infarct and previous stroke. The prevalence of atrial fibrillation was also significantly higher in Group 1 than in Group 0 (in the TOAST classification, strokes classified as cardioembolic were less numerous than the cases of atrial fibrillation, because some cases of atrial fibrillation had been classified as undetermined, being associated with other possible causes of stroke).

Thus, Group 1 included many strokes associated with atrial fibrillation and large vessel atherosclerosis, all conditions that tend to generate large ischemic cerebral lesions, thus determining an important acute stress condition. Transient hyperglycemia, accentuated by the possible presence of prediabetes, could be the consequence and the indicator of this stress condition.

### Is it important to normalize stress hyperglycemia in stroke patients?

As illustrated in the previous paragraphs, the results of this study seem to indicate that hyperglycemia in non-diabetic stroke patients is primarily a consequence, and not a cause, of stroke severity. This would lead to deny the usefulness of an aggressive treatment of hyperglycemia in the acute phase of stroke. However, it is necessary to remember that this cross-sectional study, like all cross-sectional studies, does not have the power to establish with certainty the relationships of cause and effect. In addition, controlling hyperglycemia may reduce the risk of hemorrhagic transformation of ischemic lesions. Finally, there is also the possibility that hyperglycemia may further worsen the evolution of an already severe stroke.

Gentile et al. [40] in 2006 showed that, among 373 hyperglycemic patients with ischemic stroke, those who achieved a normalization of blood glucose in 48 h had a lower mortality than patients with persistent hyperglycemia (the latter, however, had much higher baseline blood glucose levels). The retrospective design of the study and the lack of control, in the analysis, for the initial glucose levels, do not allow to exclude that the persistently hyperglycemic patients were already the most severe ones from stroke onset. Similarly, in 25 ischemic stroke patients who underwent continuous glycemic monitoring, Baird et al. [41] found that those with persistently elevated glucose levels had a significant tendency to cerebral infarct expansion. However, this study did not allow to establish what was the cause and what the effect between persistent hyperglycemia and infarct expansion.

Overall, the intervention studies carried out so far are not in favor of an aggressive treatment of stress hyperglycemia in patients with acute stroke [42]. For example, the GIST-UK study [43], concerning glucose-potassium-insulin infusion, showed no significant clinical benefit. More recently, the INSULINFARCT trial [44] showed that the brain infarcts intensively treated with insulin were larger than the infarcts treated with the usual dose of subcutaneous insulin. However, hyperglycemia was not required for inclusion in this study.

With a recommendation class IIA (moderate) and a level of evidence C-LD (limited data), current guidelines [45] consider reasonable to treat hyperglycemia to keep it in the range of 140–180 mg/dl (so treatment would only be recommended for glucose levels higher than 180 mg/dl), closely monitoring blood glucose to avoid hypoglycemia.

### Previous studies on the subject

In the early 90s some small studies denied the importance of “stress hyperglycemia” as a worsening factor in acute stroke [1–3]. In a larger study, Roquer et al. [4] in 2015 came to the conclusion that “the relationship between hyperglycemia and poor outcome reflects stress response, rather than a deleterious effect of glucose”. More recently, in 790 patients with ischemic stroke Tziomalos et al. [5] found that stress hyperglycemia was associated with mortality and disability only when the analysis was not adjusted for NIHSS score.

On the contrary, many other studies have suggested a direct unfavorable effect of stress hyperglycemia on stroke outcomes. In 1997, in 750 non-diabetic patients with acute stroke, Weir et al. [6] found that “raised plasma glucose predicts a poor prognosis after correcting for age, stroke severity and stroke subtype, which is therefore unlikely to be solely a stress response and should arguably be treated actively”. In recent years, numerous studies mainly from Chinese authors have stressed the importance of using the blood glucose/glycated hemoglobin ratio as an indicator of stress hyperglycemia (SHR) [7–13]. In particular, stroke patients were divided into quartiles according to SHR [7–10]: after correction for various possible confounding factors (not including, however, cerebral lesion size), the patients in the high quartile had a worse outcome than those in the low quartile.

### Limitations

The main limitation of this study stems from its retrospective nature, although a large sample like ours would have hardly been achieved in a prospective study. However, an ad hoc prospective study, with seriate glycemic measurements, would have allowed to precisely define the glycemic curves, with maximum values and durations, while in this study it was only possible to evaluate the fasting blood glucose at a specific time (the morning after admission).

In addition, the lack of glycated hemoglobin measurements in non-hyperglycemic non-diabetic patients does not allow us to exclude that some non-hyperglycemic diabetic patients may have been incorrectly classified in Group 0. However, any such errors would have been diluted in the large number of Group 0 patients.

Unfortunately, the most relevant group, Group 1, was also the smallest group of this study, despite the large total number of strokes considered. However, this did not prevent highly significant differences, compared to the other groups, from being obtained, in line with what is known from literature data.

Finally, due to the lack of glycated hemoglobin in group 0 we could not use the blood glucose/glycated hemoglobin ratio [7–10] as an indicator of stress hyperglycemia. However, the division of the sample into the four groups defined by the presence/absence of diabetes and the presence/absence of hyperglycemia might provide more useful information than the division of the sample into four quartiles according to blood glucose/glycated hemoglobin ratio.

## Conclusions

This study has confirmed that, among acute stroke patients, the hyperglycemic non-diabetic ones have the worst prognosis. However, this group was not independently associated with disability and mortality if stroke severity, cerebral lesion size and acute phase markers were simultaneously accounted for. Only decompensated diabetic patients had a poor prognosis independently of these factors. Overall, hyperglycemia in non-diabetic patients appears to be mainly an epiphenomenon of most severe strokes, and for this reason its aggressive treatment might not be necessary.

## Data Availability

The data that support the findings of this study are available from the corresponding author upon reasonable request.

## Abbreviations

CLD: Cerebral lesion diameter
CRP: C-reactive protein
HbA1c: Glycated hemoglobin
HDL: High density lipoprotein
LACS: Lacunar syndrome
mRS: Modified Rankin scale
NIHSS: National Institutes of Health Stroke Scale
OCSP: Oxfordshire Community Stroke Project
PACS: Partial anterior circulation syndrome
POCS: Posterior circulation syndrome
TACS: Total anterior circulation syndrome
TOAST: Trial of Org 10172 in Acute Stroke Treatment
